# Pear‐Shaped Eggs Evolved to Maximize the Surface Area‐to‐Volume Ratio, Increase Metabolism, and Shorten Incubation Time in Birds

**DOI:** 10.1111/1749-4877.12936

**Published:** 2024-12-29

**Authors:** Valeriy G. Narushin, Michael N. Romanov, Darren K. Griffin

**Affiliations:** ^1^ Research Institute for Environment Treatment Zaporizhya Ukraine; ^2^ Vita‐Market Ltd Zaporizhya Ukraine; ^3^ School of Natural Sciences Canterbury Kent UK; ^4^ L. K. Ernst Federal Research Center for Animal Husbandry Dubrovitsy, Podolsk Moscow Oblast Russia; ^5^ Animal Genomics and Bioresource Research Unit (AGB Research Unit), Faculty of Science Kasetsart University Chatuchak Bangkok Thailand

**Keywords:** avian eggs, egg shape, egg surface area, egg volume, metabolic rate, pyriform eggs, S‐to‐V ratio

## Abstract

Bird eggs can be spherical, ellipsoid, ovoid, or pear‐shaped (pyriform), the latter being the most complex. There is however no unambiguous evolutionary/adaptive explanation for this final, exotic shape. We hypothesized that pyriform eggs have a larger surface area‐to‐volume ratio (*S*/*V*) that may be a criterion for increased embryo metabolism. By integrating mathematical approaches, we confirmed this to be the case and developed a model of the *egg metabolic rate* defined as the ratio of *S*/*V* to its maximum possible value, depending on egg length. We found this to be inversely proportional to the egg incubation period and concluded that the complex pyriform shape is most likely due to embryo metabolism increase and, as a result, a reduction in the incubation period and shortened hatching time. As a result of this study, we conclude that some avian eggs are pyriform as this may attain a larger *S*/*V* ratio making them grow and hatch quicker.

## Graphical abstract

Some eggs are pyriform as this may attain a larger surface area‐to‐volume ratio making them grow and hatch quicker.

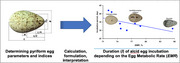

## 1 Introduction

**FIGURE 1:**
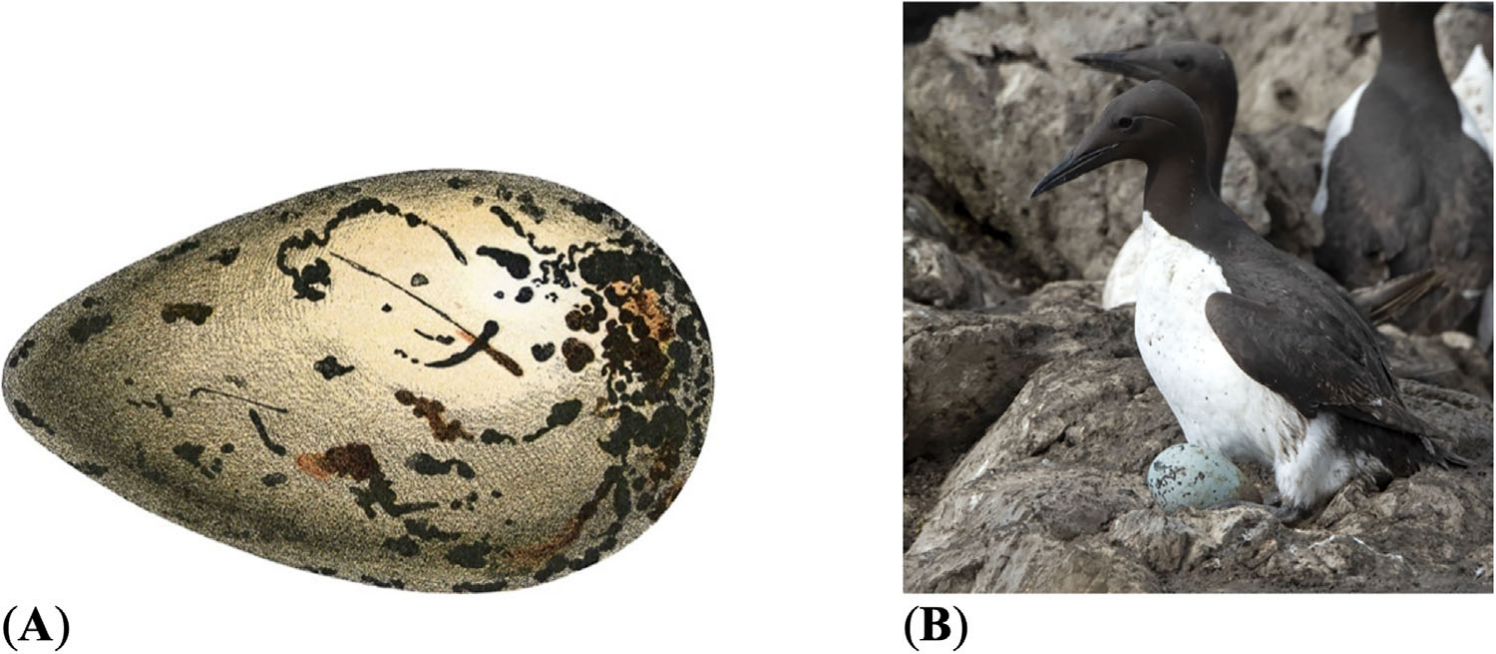
Examples of pyriform alcid eggs. (A) Egg of an extinct species of Alcidae, great auk (*Pinguinus impennis*); https://commons.wikimedia.org/wiki/File:Oeufs002b,47.png (by Adolphe Millot; public domain). (B) A common murre (*Uria aalge*) sitting on an egg at the breeding colony; https://www.usgs.gov/media/images/close‐common‐murre‐sitting‐egg‐breeding‐colony‐gull‐island (by Sarah Schoen, US Geological Survey; public domain).

There are four primary categories that can be used to characterize all profiles of eggs, namely: circular, elliptical, oval, and pyriform (pear‐shaped or conical; Nishiyama 2012). The first three types are more common in nature and have standard geometric analogs (sphere, ellipsoid, and ovoid) and the respective dependences for their mathematical description. As for the last variety, the pyriform profile (Figure 1A), its mathematical description is more complex. We (and others) have applied various mathematic means to describe this profile (Biggins, Thompson, and Birkhead 2018; Narushin, Romanov, and Griffin 2021a, 2023a, 2023c; Narushin et al. 2023), including a universal formulation for all egg shapes (Narushin, Romanov, and Griffin 2021a). Despite this, neither a formulation for pyriform eggs nor a universal formulation is currently present in specialized geometric and/or mathematical reference books. Nonetheless, their key role in completing the roster of all egg shapes has prompted an abundance of studies aimed at identifying the possible adaptive, physiological, environmental, and other benefits that influenced their evolution.

Perhaps the most pronounced pyriform egg shape is characteristic of the auk seabird family Alcidae, some of which (like the great auk) are recently extinct (Hays, Ljubičić, and Hauber 2020; Figure 1). Typical extant representative eggs of this shape are those of guillemot, which is the common name for several alcid species. A number of hypotheses pertaining to the reasons why the evolutionary process and adaptation were directed toward this particular egg shape have been proposed. Prominent among them is the increased resistance of the shell to cyclic short‐term loads. According to Birkhead et al. (2017), this may occur as a consequence of overcrowding of the community nesting on a limited area of breeding rock ledges, as a result of which, adults involuntarily step on laid eggs in the absence of the hen. The pyriform shape helps the eggs adhere better to the ground, which, to some extent, distributes the load more evenly along the surface. Since these species do not build nests and lay eggs directly on rock ledges, an alternative adaptive reason for the pyriform shape of the eggs may be to complicate their rotation and, accordingly, prevent them from rolling down on sloping surfaces (Birkhead, Thompson, and Biggins 2017, Birkhead, Thompson, and Montgomerie 2018; Hays and Hauber 2018; Figure 1B).

One more hypothesis that does not exclude, but perhaps complements, the previous ones is a shift in the center of gravity that promotes an upslope orientation of the blunt pole, making it difficult for the shell at the blunt end (where the air cell is located) to be contaminated with feces and other debris characteristic of bird colonies (Birkhead, Thompson, and Montgomerie 2018).

The conditions in which the hatching process takes place in this breeding community are rather difficult (Figure 1B). An egg, dirty with feces, which is trampled by neighbors in the colony, trying to throw it off a cliff, and even considering insufficiently comfortable climatic conditions, should nevertheless ensure the survival of the embryo. The evolutionary response to such environmental conditions is an increase in the ratio of egg surface area (*S*) to volume (*V*). This is expected because, in biology, such examples have been previously reported. Take, for instance, Allen's rule (Allen 1877), according to which the ratio of body *S* to *V* in homeothermic animals is adapted to the conditions of their habitat. This ratio is extremely important because it determines the efficiency of metabolism in the body. A larger *S*/*V* ratio means there is more surface area available for metabolism. This makes it easier for the body to absorb essential nutrients and eliminate waste products.

The *S/V* ratio in bird eggs (rather than whole organisms) and its association with metabolism are somewhat understudied, and it is not known whether a greater *S*/*V* ratio necessarily leads to higher metabolism and, ultimately, accelerated embryo growth. Atanasov (2012) demonstrated a close relationship (with *R* ^2^ = 0.931) of the *V*/*S* ratio with the lifespan of many organisms (unicellular organisms, poikilotherms, mammals, and birds). In similar studies that analyzed published egg data from various bird species, Atanasov (2019) found a similar relationship between *V*/*S* and the duration of egg incubation, albeit with a lower determination coefficient (*R* ^2^ = 0.561). In other words, it can be assumed that an increase in the value of *S*/*V*, with some degree of probability (perhaps less for eggs than for living organisms), can lead to an accelerated incubation period. Consideration of a much earlier study (Hoyt 1976), however, casts some doubt on the relationship of *S/V* ratio and the metabolic processes in the egg. Hoyt (1976) referred to the studies by Ar et al. (1974) and Wangensteen, Wilson, and Rahn (1970) that did not find a relationship between pore area, pore density, shell thickness, on the one hand, and egg *S* on the other. Hoyt (1976) concluded, “*that the functional significance of the variation in the shapes of birds’ eggs is not related to physiological exchanges with the environment*.”

In several studies (Paganelli, Olszowka, and Ar 1974; Hoyt 1976; Tatum 1977), considerable attention has been paid to the relationship between the *S* value and *V* to the power of 2/3 (i.e., *V* ^2/3^). To some extent, this interest could be explained by the possibility of mathematical calculation of *S* due to the lack of accurate direct methods for measuring this parameter. Moreover, while Paganelli, Olszowka, and Ar (1974) presented the value of this ratio in the form of a certain constant equal to 4.951, other authors make it dependent on the geometric dimensions of the egg. Hoyt (1976) used its relationship with the length‐to‐maximum breadth ratio (*L*/*B*) of the egg. Tatum (1977), in addition to this relationship, used other geometric nuances of the shape, expressed in the form of mathematical relationships that take into account the radii of curvature of various sections of the egg as was proposed by Preston (1968). This approach is very relevant and important in analytical studies related to measuring the parameters of bird eggs, and in a number of our own studies (Narushin et al. 2020a, 2022a, 2024; Narushin, Romanov, and Griffin 2021c, 2022b), we also made such attempts. As a result, a clear algorithm has been developed for recalculating the value of *S* based on the measured or computed *V* value.

Judging from the results of studies conducted to analyze the influence of the *S*/*V* ratio on indicators of various biological functions (e.g., Cohen, Moreh, and Chayoth 1999; Cragg 2016; Harris and Theriot 2018; Lewis 2023), the *S*/*V* value appears to be a very relevant index of great importance for assessing the embryonic characteristics of bird eggs as a unique evolutionary and adaptive system (Blackburn and Stewart 2021). In this regard, to expand our understanding of possible variations in the *S*/*V* ratio and its relationship with other parameters of bird eggs, we developed methodological aspects of performing experimental and theoretical studies, a detailed description of which is presented below. Specifically, we tested the hypothesis that it is the *S*/*V* ratio that is the underlying driver for the evolution of the most exotic of egg shapes, mediated by the pressure to metabolize, grow, and thus hatch more quickly.

## 2 Materials and Methods

### 2.1 Theoretical Aspects

In our previous research (Narushin et al. 2024), based on the obtained and verified mathematical model of the contours of a bird's egg (Narushin et al. 2023b), we derived calculation formulae for calculating its volume (*V*) and surface area (*S*), the ratio of which gives the dependence we are interested in:

(1) $$\frac{S}{V}=\frac{128\left(0.389+0.188\frac{B}{L}-0.063\frac{w}{L}+0.365\frac{D_{p}}{B}+0.114\frac{D_{p}}{L}-0.168\frac{w}{L}\cdot \frac{B}{L}+0.46\frac{w}{L}\cdot \frac{D_{p}}{B}+0.484\frac{w}{L}\cdot \frac{D_{p}}{L}\right)}{\left(8.917-29.998\frac{w}{L}\right){\left(\frac{D_{p}}{B}\right)}^{2}+\left(2.459+88.647\frac{w}{L}\right)\frac{D_{p}}{B}-36.26\frac{w}{L}+12.453}\cdot \frac{1}{B}$$

where *L* is the egg length, *B* is its maximum breadth, *w* is the displacement value of *B* from the center of the egg corresponding to the point *L*/2, and *D_p_* is the diameter of the egg at a point distant from the pointed end by the value of *L*/4.

**FIGURE 2:**
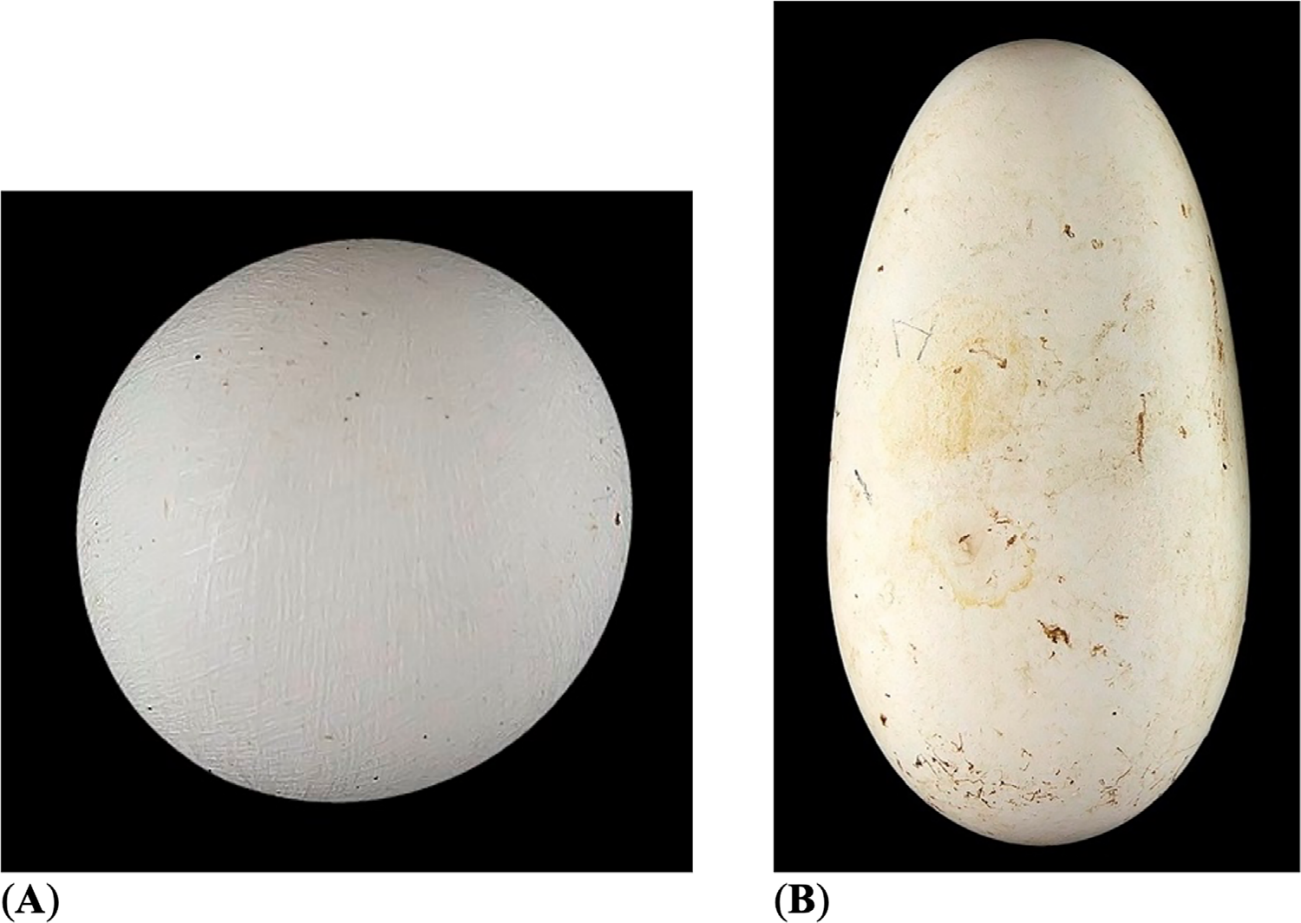
Representatives of spherical (A) and elongated (B) bird eggs. (A) Pin‐tailed whydah (*Vidua macroura*); https://commons.wikimedia.org/wiki/File:Vidua_macroura_MHNT.Z.2010.11.150.20.jpg. (B) African sacred ibis (*Threskiornis aethiopicus*); https://commons.wikimedia.org/wiki/File:Threskiornis_aethiopicus_MHNT.ZOO.2010.11.63.1.jpg (by Roger Culos, collection of Jacques Perrin de Brichambaut; CC‐BY‐SA‐4.0).

It can be argued that the *S*/*V* ratio value depends on three ratios (or indices), that is, *B*/*L*, *w*/*L*, and *D_p_*/*B*, since the *D_p_*/*L* ratio used in Equation (1) can be easily represented as a product of the corresponding indices: *D_p_*/*B*·*B*/*L*. Let us examine the boundaries of possible variation of the above indices used. The shape index (*B*/*L*) characterizes the elongation of the egg relative to the length of the axis. The uppermost value of this index is limited to 1; that is, it characterizes a spherical egg; for example, the *B*/*L* value in pin‐tailed whydah (*Vidua macroura*) is 0.99 (Figure 2A). As for the lower limit, it is usually taken equal to 0.5 in such calculations (e.g., Tatum 1977; Narushin, Romanov, and Griffin 2021a). Of the examples of the presence of such a ratio among actual eggs, the *B*/*L* value of African sacred ibis (*Macrocephalon maleo*) is not much higher than 0.5, being equal to 0.52 (Figure 2B), so it can quite reasonably be, with some degree of accuracy, approximately taken equal to 0.5.

In our previous studies (Narushin, Romanov, and Griffin 2021a, 2022b, 2023a, 2023c; Narushin et al. 2021b, 2022a, 2023b), when studying actual eggs found in nature, we tested both the theoretical limits of their variability and the degree of their adequacy. As a result, it was concluded that the *w*/*L* value that was conventionally called the displacement index of the maximum egg breadth varies in the range of 0 … 0.2.

As for the ratio *D_p_*/*B* that we dubbed the conicity index, its lower limit (denoted by the subscript pyr, i.e., inherent in pyriform eggs) characterizes the parabolic shape (Narushin, Romanov, and Griffin 2021a, 2023c; Narushin et al. 2023b) and is determined by the following previously derived formula (Narushin et al. 2022a):

(2) $${\frac{D_{p}}{B}}_{\mathrm{pyr}}=\frac{0.707}{\sqrt{1+2\frac{w}{L}}}.$$

Its upper limit (denoted with the subscript Hug, i.e., determined by the Hügelschäffer's model (as described in Narushin et al. 2020b, 2022a) is characteristic of the ovoid shape (e.g., in chicken eggs) and can be defined by the following dependence:

(3) $${\frac{D_{p}}{B}}_{\mathrm{Hug}}=\frac{0.866}{\sqrt{1+2\frac{w}{L}+4{\left(\frac{w}{L}\right)}^{2}}}.$$

Hence, Equation (1) can be represented as follows:

(4) $$\frac{S}{V}=\frac{K}{B},$$

where *K* is a certain coefficient determined by the dependence:

(5) $$K=\frac{128\left(0.389+0.188\frac{B}{L}-0.063\frac{w}{L}+0.365\frac{D_{p}}{B}+0.114\frac{D_{p}}{L}-0.168\frac{w}{L}\cdot \frac{B}{L}+0.46\frac{w}{L}\cdot \frac{D_{p}}{B}+0.484\frac{w}{L}\cdot \frac{D_{p}}{L}\right)}{\left(8.917-29.998\frac{w}{L}\right){\left(\frac{D_{p}}{B}\right)}^{2}+\left(2.459+88.647\frac{w}{L}\right)\frac{D_{p}}{B}-36.26\frac{w}{L}+12.453}.$$

Thus, considering the range of variability of the three said indices, changes in the *K* coefficient value (Equation 5) can be presented in the form of graphical dependences, which we conventionally divided into two subtypes: *K*_pyr_ for conical eggs (when the conicity index is determined according to Equation 2), and *K*_ov_ for ovoid eggs (when the conicity index is defined by Equation 3). These results are shown in Figure 4.

**FIGURE 3:**
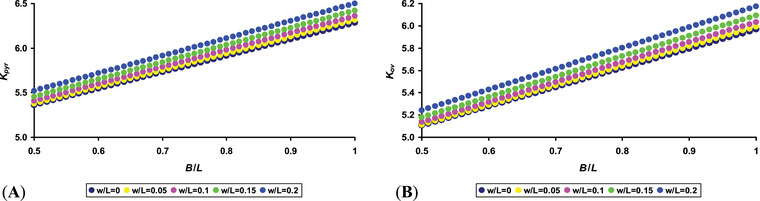
Graphical interpretation of changes in the *K* coefficient value (Equation 5) for conical eggs, *K*_pyr_ (A), and for ovoid eggs, *K*_ov_ (B).

Graphic visualization (Figure 3) demonstrates that the *K*_pyr_ values exceed those of *K*_ov_ at the same values of *B*/*L* and *w*/*L* indices. However, it is quite difficult to say unequivocally that this proportionally affects the *S*/*V* ratio. This uncertainty lies in the fact that parameter *B* is located both in the numerator and in the denominator of Equation (1). Thus, increasing this parameter can either increase or automatically decrease the final value of the *S*/*V* ratio.

For a deeper analysis of the relationships and functional influence of the egg parameters included in Equation (1) on the value of embryonic metabolism, we suggested simulation studies by creating a database of virtual eggs of various shapes and sizes that exist in nature and correspondingly calculating the values of their *S*/*V* ratios.

One can also suggest that a possible increase in the *S*/*V* value may be a response of the bird not only to harsher environmental conditions but also to the egg incubation period. That is, increasing the metabolic processes of the developing embryo can help reduce the time of its maturation, according to the premise suggested by Atanasov (2019). This hypothesis can be tested by comparing pyriform and regular ovoid eggs. Considering that the egg incubation time is directly influenced by egg weight (Rahn and Ar 1974), the goal of comparative assessment of two types of egg profiles can be reduced to the development of some kind of universal methodological approach that makes it possible to evaluate eggs with obvious differences not only in shape and relationships of geometric parameters but also in weight. Without a doubt, the possibility of using lower energy costs for incubation may be one of the fundamental reasons for reducing the incubation period. One of the possibilities for such a reduction is to give the egg a pear shape, which will increase the surface area consuming heat relative to its volume. It is this fact that we wanted to highlight with our work. Unfortunately, we were unable to carry out field studies directly on the clutches of Alcidae birds, by directly measuring heat exchange. In addition to the ethical component of protecting animals from unnecessary manipulative experiments, an important restraining factor was the need to place all groups of the species under the same environmental conditions. Otherwise, the data obtained will not allow for their relevant comparison and evaluation. In this regard, we limited ourselves to a theoretical approach to obtaining the necessary information, based on data from the classical laws of mathematics, geometry, and physics.

Therefore, another goal of our experimental research was to develop such a technique and test the hypothesis that, of two eggs, the one with the higher *S*/*V* ratio has a shorter period of embryonic development.

### 2.2 Experimental Procedure

In the first stage, a database of virtual eggs was compiled and used to evaluate the correlation and functional relationships of the parameters included in the basic Equation (1). First, since *L* may be a more functional (stable) parameter than *B*, it was decided to slightly modify Equation (1), presenting it as follows:

(6) $$\frac{S}{V}=\frac{128\left(0.389+0.188\frac{B}{L}-0.063\frac{w}{L}+0.365\frac{D_{p}}{B}+0.114\frac{D_{p}}{B}\cdot \frac{B}{L}-0.168\frac{w}{L}\cdot \frac{B}{L}+0.46\frac{w}{L}\cdot \frac{D_{p}}{B}+0.484\frac{w}{L}\cdot \frac{D_{p}}{B}\cdot \frac{B}{L}\right)}{\left[\left(8.917-29.998\frac{w}{L}\right)\;{\left(\frac{D_{p}}{B}\right)}^{2}+\left(2.459+88.647\frac{w}{L}\right)\frac{D_{p}}{B}-36.26\frac{w}{L}+12.453\right]L\cdot \frac{B}{L}}.$$

To generate the database of virtual eggs, with a set of parameters that can presumably exist in nature, we need to determine the range of possible values for the following parameters: *B*/*L*, *w*/*L*, *D_p_*/*B*, and *L*. Based on the premises noted in the above theoretical section, the following ranges and increments of changes in the values of the indices included in Equation (6) were adopted:

*B*/*L* = [0.5 … 1.0], with an increment of 0.1;

*w*/*L* = [0 … 0.2], with an increment of 0.05.

As for the conicity index (*D_p_*/*L*), its lower limit is determined by Equation (2) and its upper limit, respectively, by Equation (3). Both boundary limits depend on the specific value of the *w*/*L* ratio. Therefore, intermediate values of *D_p_*/*B* were selected depending on the calculated minimum and maximum values with an approximate increment of 0.015…0.02. This approach provided us with 7 intermediate *D_p_*/*B* values for each corresponding *w*/*L* value. Thus, we had at our disposal sets of six *B*/*L*, five *w*/*L*, and nine *D_p_*/*B* values, the various ratios of which provided 891 combinations of values characteristic of the entire variety of bird eggs existing in nature.

In addition to the above‐noted indices, Equation (6) includes the *L* value that was also considered based on possible variations in its values ranging between 1 cm (e.g., in hummingbird eggs; Ornelas 2010) and 15 cm (e.g., in African ostrich eggs; Kokoszyński 2017). Thus, in the virtual egg database to calculate the *S*/*V* ratio, we used the values of *L* = [1 … 15] cm with an increment of 2 cm.

In the next step, we assessed possible differences in egg incubation periods and their relationship with metabolic rate. We decided to focus on studying this dependence on the eggs of birds of the same family Alcidae. This approach is justified by the fact that the proximity of the anatomical structure of birds, their living conditions, the geometric and physical dimensions of their eggs, as well as the biological and physiological nuances of incubation, will allow, if not eliminate, then at least minimize possible doubts about the reliability of the results obtained. Although most genera of Alcidae are characterized by a pyriform (conical) shape of eggs, nevertheless, according to Hays, Ljubičić, and Hauber (2020), not all representatives of this species adhere to the geometric rule of conicity when forming an egg. Although the eggs they lay are also elongated, their pointed end is somewhat oval and does not fully correspond to the pyriform category, whereas it is also difficult to classify it as a classic oval. Such differences were quite useful for testing our hypothesis.

Departing from the cladogram of the Alcidae family (Smith 2011), we used open sources to select the available data on eggs (i.e., their images linked to a metric scale) laid by representatives of this family and the duration of their incubation. Naturally, available images of egg profiles of typical representatives of a particular species can provide only limited information for analysis, due to their small number. However, such inaccuracy should not globally affect the possible confirmation or refutation of our hypothesis. After all, intraspecific variation in egg shape is not as variable as interspecific variation (e.g., Petersen 1992; Barta and Székely 1997; Mónus and Barta 2005; Englert Duursma et al. 2018; Hays, Ljubičić, and Hauber 2020; Liu et al. 2023).

As a result, the following database was formed from the selected data sources (Table 1), which served as the basis for further measurements and calculations.

**TABLE 1:** Initial data for the analysis of alcid eggs.

##	English (Latin) name	Incubation period, *I* (days)	Ref. of information about *I*	Image source/reference (number of images)
1	Razorbill (*Alca torda*)	34	Wagner (1991)	https://www.charlestonmuseum.org/research/collection/?tax_family=Alcidae&page=1 (2)
2	Common murre (*Uria aalge*)	31	Bennett (2001)	https://www.charlestonmuseum.org/research/collection/?tax_family=Alcidae&page=1 (7)
3	Thick‐billed murre (*Uria lomvia*)	32.5	Thalathara (2013)	https://www.charlestonmuseum.org/research/collection/?tax_family=Alcidae&page=1 (4)
4	Pigeon guillemot (*Cepphus columba*)	31	Hauf (2021)	https://www.charlestonmuseum.org/research/collection/?tax_family=Alcidae&page=1 (4)
5	Marbled murrelet (*Brachyramphus marmoratus*)	30	Smart (1999)	https://staff.washington.edu/puffinus/brachyramphus‐marmoratus/ (2)
6	Ancient murrelet (*Synthliboramphus antiquus*)	34	Zimmermann and Hipfner (2007)	https://www.charlestonmuseum.org/research/collection/?tax_family=Alcidae&page=1 (2)
7	Cassin's auklet (*Ptychoramphus aleuticus*)	38	Zimmermann and Hipfner (2007)	https://www.charlestonmuseum.org/research/collection/?tax_family=Alcidae&page=1 (4)
8	Rhinoceros auklet (*Cerorhinca monocerata*)	42	Alaska SeaLife Center (2023)	https://www.charlestonmuseum.org/research/collection/?tax_family=Alcidae&page=1 (1)
9	Tufted puffin (*Fratercula cirrhata*)	41	Kaufman (2024)	https://www.charlestonmuseum.org/research/collection/?tax_family=Alcidae&page=1 (4)

**FIGURE 4:**
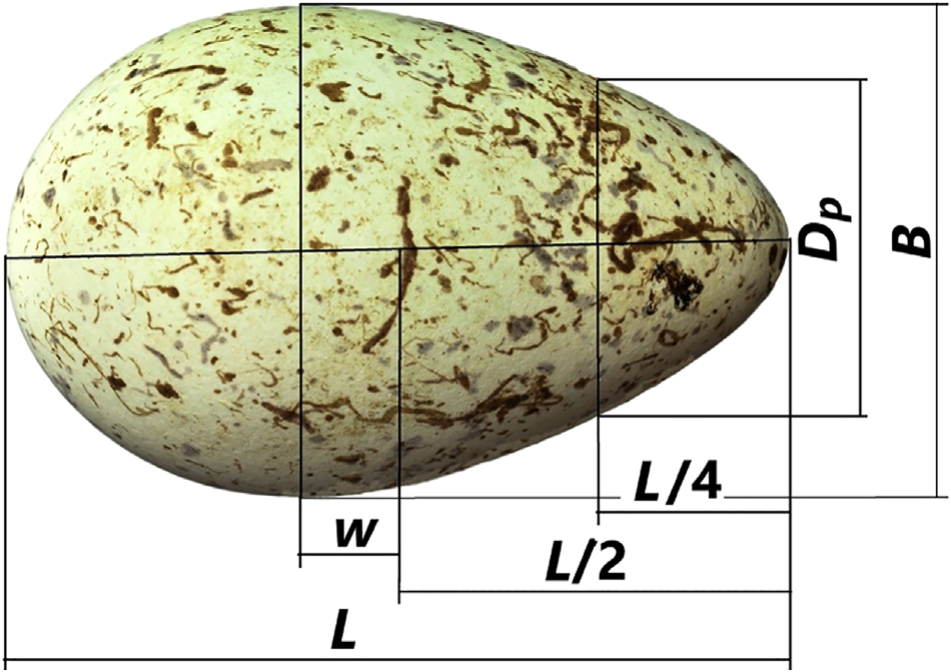
Schematic representation of the measured geometric parameters using the example of an image of a thick‐billed murre egg (*Uria lomvia*); https://commons.wikimedia.org/wiki/File:Uria_lomvia_MWNH_2182.JPG (by Klaus Rassinger and Gerhard Cammerer, Natural History Collections of the Museum Wiesbaden; CC‐BY‐SA‐3.0).

Each egg image chosen for subsequent analysis was examined as follows. Measurements of geometric parameters were carried out in pixels using the Microsoft Office Picture Manager program. We measured *B*, *L*, *D_p_*, and *w*. Using the ruler with metric units presented next to the image, we made the appropriate conversion of pixels to centimeters for all measured geometric parameters. A schematic representation of the measured geometric parameters, using the example of an image of a thick‐billed murre (*Uria lomvia*) egg, is presented in Figure 4.

To process the results, we used statistical and mathematical algorithms available in the STATISTICA 5.5 program (StatSoft, Inc./TIBCO, Palo Alto, CA, USA), as well as applications in the Microsoft Excel program. Herewith, the validity of the obtained relationships was assessed by the value of the Pearson correlation coefficient (*R*), and that of regression models by the coefficient of determination (*R* ^2^) with confirmation of their significance at the level of *p* < 0.05.

## 3 Results and Discussion

### 3.1 Simulation

As we stated in the Theoretical Aspects section (see Materials and Methods), the *S*/*V* ratio value is somewhat influenced by the following three indices: *B*/*L*, *w*/*L*, and *D_p_*/*B*, as well as *L*, where *B* is the maximum egg breadth, *L* is its length, *w* is the displacement value of *B* from the center of the egg, and *D_p_* is the diameter of the egg at a point distant from the pointed end by the value of *L*/4. The degree of influence of these indices can be determined by quantitatively measuring the effect of their impact on *S*/*V* using regression analysis (e.g., Petchko 2018).

The resultant regression equation for any bird egg, the length of which is *L* = 1…15 cm, had the following view:

(7) $$\frac{S}{V}=\left(16.544-8.431\frac{B}{L}-0.995\frac{w}{L}-3.051\frac{D_{p}}{B}\right)\cdot \frac{1}{L},$$

with *R* ^2^ = 0.963 (*p* < 0.05).

**FIGURE 5:**
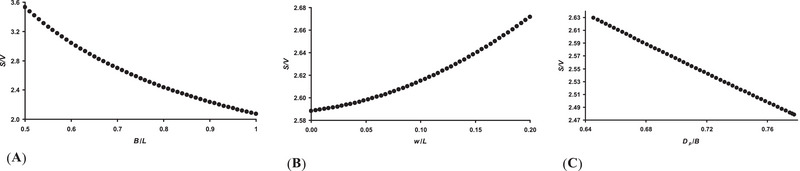
Visualization of possible functional changes in the *S*/*V* value depending on the values of the *B*/*L* (A), *w*/*L* (B), and *D_p_*/*B* (C) indices.

As follows from Equation (7), the largest coefficient value in the regression equation refers to the egg shape index value (*B*/*L*), followed by the conicity index (*D_p_*/*B*). The displacement index of *B* (*w*/*L*) has the least influence. Most likely, the functional component of the latter index in the considered general structure of mathematical relationships between the level of metabolism of bird eggs (*S*/*V*) is of an auxiliary nature since it directly depends on the *D_p_*/*B* values (Equations 2 and 3). To visualize the degree of influence of each of the indices on *S*/*V*, we carried out a conditional mathematical calculation using Formula (7), alternately selecting certain averaged values of the initial parameters: *B*/*L* = 0.74, *w*/*L* = 0.1, and *D_p_*/*B* = 0.7 for the length of a conventional egg *L* = 3 cm (Figure 5).

Judging from the results obtained, we can suggest, purely mathematically, that the mother bird has several ways to increase the metabolic level of the embryo, including the *S*/*V* ratio. This is possible by lowering the values of the *B*/*L* (Figure 5A) and *D_p_*/*B* (Figure 5C) indices, and/or a corresponding increase in *w*/*L* (Figure 5B). One can effectively vary the *B*/*L* value, based on the correlation analysis; however, this is obviously not very functional, since *L* and *B* are largely determined by the anatomical features of the bird (e.g., Birkhead et al. 2019). Not without reason, many studies demonstrate fairly stable shape index values within species (e.g., Petersen 1992; Barta and Székely 1997; Mónus and Barta 2005; Englert Duursma et al. 2018; Hays, Ljubičić, and Hauber 2020; Liu et al. 2023). However, it is worth paying close attention to those species that “specialize” in laying pyriform (conical) eggs and thereby reduce this indicator. For example, in our previous studies (Narushin et al. 2022c), the average *B*/*L* value of pyriform eggs was 0.70, while it was 0.74 for ovoid eggs.

As for *D_p_*/*B*, its reduction can aid the hen in increasing the metabolism of the embryo it produces. As we have already noted, it is inappropriate to increase the *B* value, since this leads to the opposite effect; however, decreasing the size of *D_p_* looks like a completely logical and feasible process. As a result, the shape evolution and adaptation of eggs laid in stressful conditions such as cliff edges could be directed toward giving a more pyriform shape to the egg. We have already noted that the lower (i.e., most cone‐shaped) limit inherent in pyriform eggs characterizes the parabolic shape (Narushin, Romanov, and Griffin 2021a; Narushin et al. 2023b, 2024) and is determined by Equation (2) according to Narushin et al. (2022a). This formula also establishes a mathematical relationship between two indices (*D_p_*/*B* and *w*/*L*). Thus, a mother bird attempting to create eggs of pyriform shape (i.e., to reduce *D_p_*/*B*) has to increase *w*/*L* because this value is in the denominator of Equation (2). The *w*/*L* ratio can be increased only by correspondingly increasing *w*, that is, by moving the *B* axis as far as possible from the center. Naturally, this also poses limitations. Purely geometrically, this distance cannot exceed half the length of the egg. However, based on the characteristics of the structural components of the egg contents, it is necessary to leave sufficient volume for the air chamber, which should increase during incubation, and provide for the presence of a chalaza that holds the yolk. In other words, the bird does not have the opportunity to evolve toward laying eggs that have the shape of a classic cone, since the structure of the egg will be subject to fundamental alteration. Accordingly, such a reshaping will, in no way, contribute to the vital functions of the embryo. However, despite the physiological complexities and geometric limitations of the biological design of the egg, the bird does everything possible to use the opportunity to increase the *w*/*L* value effectively, which results in its average values being much higher in pyriform eggs than in ovoid ones. In our previous investigation (Narushin et al. 2022c), the average *w*/*L* value of pyriform eggs was 0.12, while that of ovoid eggs was 0.06. If we present Equation (2) in the following form:

(8) $$D_{p}=L\cdot \frac{B}{L}\cdot \frac{0.707}{\sqrt{1+2\frac{w}{L}}},$$

we can conclude that the *D_p_* value, in addition to *w*/*L*, is also directly influenced by *B*/*L*. Thus, from the viewpoint of basic mathematics, reducing the size of the diameter *D_p_*, that is, giving the egg a more pyriform shape, is possible due to a corresponding decrease in the *B*/*L* value, since this index is located in the numerator of Equation (8) and an increase in *w*/*L* located in the denominator.

Thus, to increase the level of metabolism of the egg and, accordingly, increase the *S*/*V* ratio, the mother's body can only provide the egg with a pyriform shape, the criterion of which can be considered the possibility of reducing the diameter (*D_p_*) at the point corresponding to *L*/4 from the pointed end. Considering that the concave shape of the egg is, in no way, acceptable, the minimum possible decrease in the *D_p_* value causes corresponding structural changes. The latter implies the maximum possible decrease in the *B*/*L* ratio and the maximum possible increase in the *w*/*L* ratio. That is, the pyriform shape principle may be a fundamental factor in the evolution and adaptation of the egg shape to increase the viability and efficiency of the embryo development within that egg.

### 3.2 Development of a Methodology for Assessing Eggs Based on Their S/V Ratio

**FIGURE 6:**
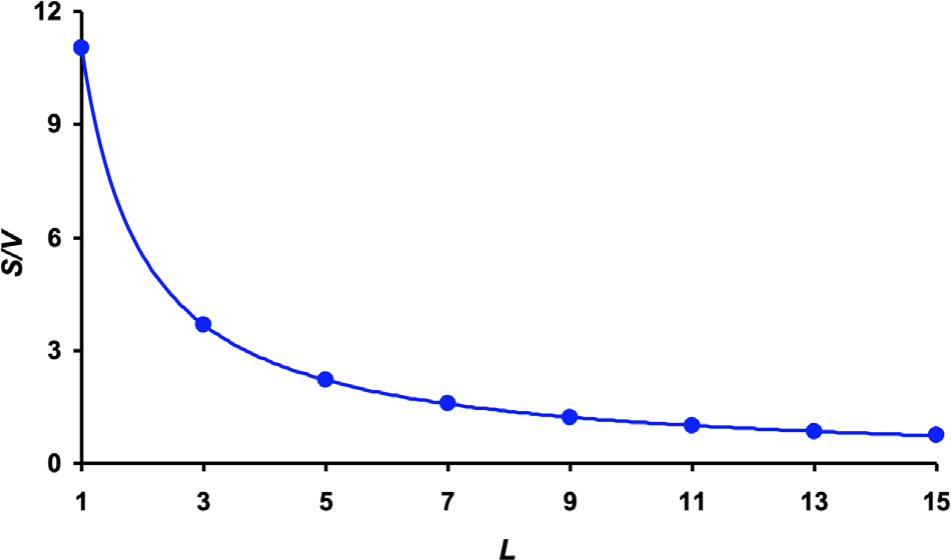
Relationship between maximum *S/V* values depending on egg length, *L*.

An important influence on the *S/V* value is exerted by *L* located in the denominator (Equation 8), in that a larger *L* reduces the *S/V* value. As a result, it is incorrect to equate the metabolic rate of two eggs of different lengths. For example, as a result of calculating our simulation model, we visualized the relationship between the maximum *S/V* values depending on the *L* value (Figure 6).

Thus, each specific egg of a certain *L* is characterized by a certain conditional maximum possible value (*S/V*_max_) that, based on the graphical dependence in Figure 6, can be approximated by the following equation:

(9) $${\frac{S}{V}}_{\max}=\frac{11.043}{L}.$$

**FIGURE 7:**
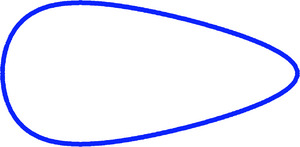
Contour of a virtual bird's egg, the geometric indices of which have the following values: *B*/*L* = 0.5, *w*/*L* = 0.2, and *D_p_*/*B* = 0.598.

Considering the nature of the dependences in Figure 5 and values from the database for our simulation model, it is relatively straightforward to validate the data in Equation (9) after substituting the index values *B*/*L* = 0.5 and *w*/*L* = 0.2 and, thereafter, by recalculating according to Equation (2), *D_p_*/*B* = 0.598 into Equation (6). Using a mathematical relationship for establishing almost any contour of a bird's egg (Narushin et al. 2023b), we recreated the shape of a virtual egg (Figure 7), the geometric indices of which correspond to the above values.

However, most often, there are few such eggs in nature, all of whose indices correspond to values that allow ensuring the *S/V*_max_ ratio for a specific *L*. The specific *S/V* values of their actual counterparts are clearly somewhat lower than the maximum. Thus, we proposed to evaluate how much the *S/V* value (Equation 6) of a particular egg, with length *L*, is less than the *S/V*_max_ value calculated according to Equation (9). More precisely, what is the percentage of the real *S/V* value in the structure of the *S/V*_max_ value? We called the value of this percentage ratio *egg metabolic rate* (EMR), which, after dividing Equation (6) by Equation (9) and multiplying by 100%, will have the following form:

(10) $$\mathrm{EMR}=\frac{0.362+0.175\frac{B}{L}-0.059\frac{w}{L}+0.34\frac{D_{p}}{B}+0.106\frac{D_{p}}{B}\cdot \frac{B}{L}-0.156\frac{w}{L}\cdot \frac{B}{L}+0.428\frac{w}{L}\cdot \frac{D_{p}}{B}+0.45\frac{w}{L}\cdot \frac{D_{p}}{B}\cdot \frac{B}{L}}{\left[\left(0.716-2.409\frac{w}{L}\right)\;{\left(\frac{D_{p}}{B}\right)}^{2}+\left(0.197+7.119\frac{w}{L}\right)\frac{D_{p}}{B}-2.914\frac{w}{L}+1\right]\frac{B}{L}}\cdot 100.$$

### 3.3 Incubation Period

**FIGURE 8:**
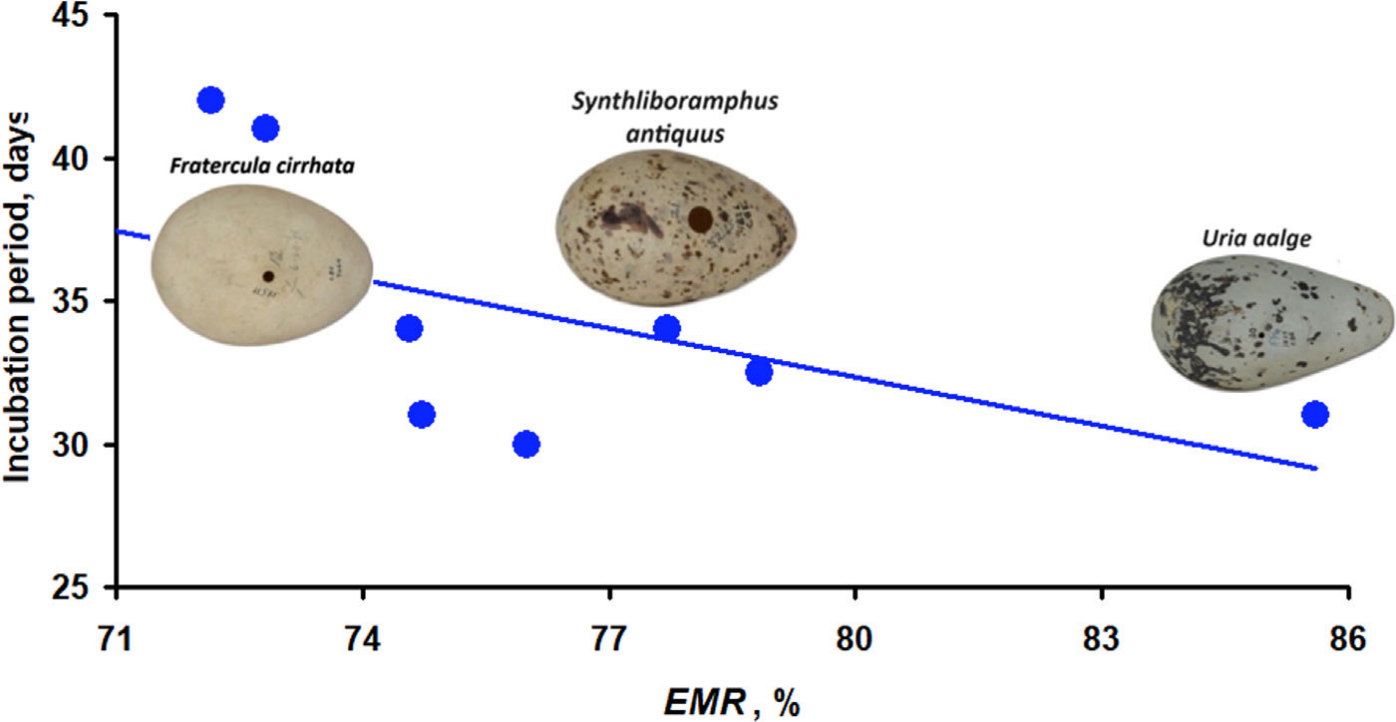
Dependence of the duration (*I*) of alcid egg incubation on the egg metabolic rate (EMR). The egg images used are courtesy of the Alcidae egg collection website, The Charleston Museum, SC; https://www.charlestonmuseum.org/research/collection/?tax_family = Alcidae&page = 1.

Having the developed methodology that can be used to indirectly estimate the EMR of eggs of any shape and size, as well as data on the incubation period (*I*) of eggs of the Alcidae family (Table 1), we can begin to analyze the possible relationship of these values. A visualization of *I* versus EMR is shown in Figure 8.

Despite some variation around the trend line (Figure 8), the inverse relationship is quite clear, demonstrating that an increase in EMR in alcids leads to a decrease in the time spent by the bird for incubating eggs (*R* = −0.632, *p* < 0.05). Hereby, the shape of the eggs indicates a corresponding transformation of their contours from the classic ovoid (in eggs with a maximum incubation period) to pyriform eggs. The latter is characteristic of those species that, as a result of evolutionary change and adaptation, managed to significantly reduce the incubation period.

It is possible that, in addition to the EMR value, the egg weight (*W*) also affects the incubation period. Indeed, it was this parameter that was used as a fundamental one in the work of Rahn and Ar (1974) to calculate *I*. Using data on 475 species of birds, those authors obtained the following allometric relationship:

(11) $$I=12.03W^{0.217},$$

with *R* = 0.86, where *I* is measured in days and *W* in grams.

Previously, Deeming et al. (2006) examined a number of scientific prerequisites for refining the possible allometric prediction of incubation time from the *W* value. In their opinion, the taxon of birds has a special influence on the *I* value. In this regard, they presented graphical dependences of *I* on *W* for eggs of different families. Despite the increase in prediction accuracy, the availability of the directly measured *W* value to perform further calculations remained mandatory.

Unfortunately, there were no data on *W* of eggs, the images of which we used to calculate the EMR. Therefore, to check the adequacy of Equation (11) for the eggs we selected, we used the results of allometric relationships obtained by Paganelli, Olszowka, and Ar (1974). Using the formula they presented for calculating the density of an egg, it was possible to obtain an approximate calculation of the *W* value depending on *V*:

(12) $$W=1.038V^{1.006}$$

where *W* is measured in grams and *V* in cm^3^.

Given the *V* values calculated for our sampled images of alcid eggs using the formula from Narushin et al. (2024) that we used to derive the equation for *S/V* (Equation 1), we calculated the *W* values using Equation (12), and, accordingly, *I* by Equation (11). However, the calculated results obtained were far from being real: the correlation between them was −0.096 and was insignificant. Such discrepancies are caused by the fact that eggs within one family, in our case Alcidae, have a range in *W* (32…133 g) significantly less than the values (0.5…1000 g) used by Rahn and Ar (1974) when deriving their dependence (Equation 11). In this regard, the relationship we obtained between *I* and EMR (Figure 8) looks much more accurate. However, we decided to evaluate the possibility of developing a more accurate prediction of *I* values for alcid eggs if, in addition to EMR, *W* values were also included in the calculation. As a result, the following relationship was obtained:

(13) $$I=103000\frac{W^{0.15}}{\mathrm{EM}R^{2}},$$

with *R* = 0.774 (*p* < 0.05), where *I* is measured in days, *W* in grams, and *EMR* in %.

Using Equation (13), the prediction of the alcid egg incubation period was significantly improved.

For the sake of discussion, we would like to admit that the duration of incubation is influenced by a whole group of factors. Some of these factors are quite trivial and have been studied to a sufficient extent. Some factors have not been given due attention, for example, a possible influence of egg pigmentation. Undoubtedly, a set of such factors and parameters structures the basis for forming the *I* indicator. We, indeed, have touched upon only one of these parameters, that is, the *S*/*V* ratio. This index, despite its logic, was mainly overlooked, for some reason, by other researchers in the field who analyzed this issue. We have managed to find only one such study (Atanasov 2019), the results of which served as a starting point for our current study. We admit that this parameter (*S*/*V*) may not be a key cause but only a consequence of other factors, such as improved heat exchange and/or air exchange, or, perhaps, a slightly larger specific area of the pigment on the shell. It is possible that even such a parameter as the change in the contact area with the substrate on which the eggs are placed also depends on the surface area of the egg, and it is quite possible that these factors are the reason for the mother bird to give the eggs a pear‐shaped form. One way or another, it seems that the *S*/*V* index can serve as a kind of integral indicator that has absorbed a certain set of parameters that affect not only the acceleration of embryonic metabolism but also a number of other factors that depend on the values of *S* and *V*, which, as a result, affects the reduction of incubation period.

### 3.4 Future Studies

The developed methodological and theoretical approaches create a reliable and promising basis for future research. First of all, this concerns a revision of the concept of predicting the egg incubation period. Herewith, it is advisable to review the entire range of bird eggs. The allometric relationship obtained by Rahn and Ar (1974) using *W* is only suitable for gross estimation. As we saw from the example of alcid eggs, the prediction of *W* within one family turned out to be extremely inaccurate. A much more promising goal might be to use *W* and EMR together in this kind of dependence. To implement such studies, in addition to the images and geometric dimensions of the eggs involved in the experiments, it is also necessary to have their *W* values.

This study area holds considerable promise for applied research, for example, in the field of industrial poultry farming. Despite the success achieved by breeders in creating fast‐growing chicken crosses, the incubation time of chicken eggs remains unchanged at 21 days. The economic benefit of reducing this period by at least 1 day would be enormous (and beyond the scope of this study to calculate). Similarly, the energy saving by hatcheries around the globe would be huge. Perhaps, to address this, a change in just a few geometric parameters of the chicken egg, for example, the *B* and *D_p_* values, to make commercial eggs a little narrower and more pear‐shaped could achieve this. If we take as a basis a standard chicken egg (Romanoff and Romanoff 1949), with *L* = 5.7 cm, *B* = 4.2 cm, and *W* = 58 g, then, a reverse recalculation is possible using Formula (13), taking the *I* value of 20 days, for instance. The only assumption, in this case, is that Equation (13) that we obtained for alcid eggs is adequate for the entire diversity of bird eggs. Naturally, this assumption is very controversial; however, it can be taken as a basis for possible research. The result of recalculation of the EMR value gives a value of 97.3%. That is, theoretically, the incubation period of chicken eggs can be reduced by 1 day if their shape is brought almost to the boundary (Figure 7) corresponding to the *S/V*_max_ value. Even if this prerequisite turns out to be unfeasible, pre‐incubation sorting of eggs according to EMR may make it possible to synchronize the hatching of chicks in selected groups.

Given the huge popularity of 3D bird egg models in engineering (Zhang et al. 2017a, 2017b, 2019, 2021, 2022; Levine, Kaplun, and Ribak 2022; Narushin et al. 2022d; Songsheng and Wang 2023) and building structures (Petrović, Obradović, and Mijailović 2011; Maulana, Yunus, and Sulistyaningrum 2015; Juračka et al. 2023; Obradović and Martinenko 2023), the approach of choosing the optimal *S/V* ratio has the potential to become a new paradigm in a range of biology disciplines and human endeavor.

## 4 Conclusions and Remarks

Analysis of the theoretical and experimental results presented herein allowed us to formulate the following main conclusions:

1. We provide compelling evidence that the most recent and complex stage in avian egg evolution, the pyriform (conical) shape, evolved as a means of increasing metabolism to hasten hatching, rather than previously proposed explanations. At the very least, we proposed an alternative hypothesis with a clear scientific basis. In harsh environmental conditions for life, subsequent egg incubation benefits from an increase in metabolism and time to hatch. One way in which this can be achieved is an adaptive variation of the egg to a pyriform shape attained through an increase in the *S/V* ratio. Since the *S* and *V* values are geometric indicators, the use of a mathematical approach to analyze their relationship with the egg shape was the optimal solution.
2. We identified three egg indices that can be considered as basic characteristics for its geometric shape and, accordingly, the *S/V* indicator. These include the shape index (*B*/*L*), the displacement index of *B* (*w*/*L*), and the conicity index (*D_p_*/*B*). The shape index has the greatest influence on the *S/V* value. In this case, the lower the *B*/*L* value, the higher the *S/V*. However, the *B*/*L* ratio is determined by the anatomical structure of the bird and the characteristics of embryogenesis and, therefore, cannot evolve too much toward a decrease. The conicity index is the next most significant in its effect on *S/V* and, similar to the shape index, is also preferable at the lowest possible values. The *w*/*L* value directly depends on *D_p_*/*B* with a clear mathematical relationship, as a result of which it takes maximum values with corresponding minimum values of the conicity index. The boundary profile of the egg, which, with some assumption, can still be found in natural conditions and the theoretical contour of which we reproduced in Figure 7, is due to the following indices: *B*/*L* = 0.5, *w*/*L* = 0.2, and *D_p_*/*B* = 0.598. These limiting values of the indices made it possible to calculate the *S/V*_max_ value and, accordingly, to develop a methodological basis for comparing eggs according to their level of metabolism, taking into account not only differences in shape but also geometric dimensions. The evaluation criterion was named EMR and represented a quantitative assessment (in %) expressed as the ratio of the *S/V*_max_ value for a particular egg to the actual value (Equation 10).
3. Using the example of eggs belonging to different bird species of the same family, Alcidae, we demonstrated a close relationship between the incubation period and the EMR value. Thus, the acceleration of metabolic processes in pyriform eggs is intended to reduce their incubation time, which is extremely important in those unfavorable conditions due to which evolutionary and adaptive changes in the egg shape occurred. A more accurate prediction of the egg incubation period in a given family is possible by using an empirical relationship, which, in addition to the EMR value, also takes into account *W*.
4. The proposed methodological and theoretical approaches can serve as the basis for implementing a number of research avenues and applications, for example, in agriculture, architecture, and engineering.
